# Activation of phosphatidylinositol 3-kinase/AKT/snail signaling pathway contributes to epithelial-mesenchymal transition-induced multi-drug resistance to sorafenib in hepatocellular carcinoma cells

**DOI:** 10.1371/journal.pone.0185088

**Published:** 2017-09-21

**Authors:** Jiejie Dong, Bo Zhai, Weihua Sun, Fengli Hu, Hao Cheng, Jun Xu

**Affiliations:** 1 Department of General Surgery, the First Affiliated Hospital of Harbin Medical University, Harbin, Heilongjiang, China; 2 Department of Hepatobiliary Surgery, the Yuncheng Central Hospital, Yuncheng, Shanxi, China; 3 Department of General Surgery, the Fourth Affiliated Hospital of Harbin Medical University, Harbin, Heilongjiang, China; 4 Heilongjiang Medical Science Institute, Harbin, Heilongjiang, China; 5 Department of Gastroenterology, the First Affiliated Hospital of Jinzhou Medical University, Jinzhou, Liaoning, China; University of South Alabama Mitchell Cancer Institute, UNITED STATES

## Abstract

Sorafenib, an orally available kinase inhibitor, is the standard first-line systemic drug for advanced hepatocellular carcinoma (HCC), and it exerts potent inhibitory activity against epithelial–mesenchymal transition (EMT) and multidrug resistance (MDR) by inhibiting mitogen-activated protein kinase (MAPK) signaling in HCC. However, after long-term exposure to sorafenib, HCC cells exhibit EMT and resistance to sorafenib. The activation of AKT by sorafenib is thought to be responsible for the development of these characteristics. The present study aims to examine the underlying mechanism and seek potential strategies to reverse this resistance and the progression to EMT. Sorafenib-resistant cells showed increased metastatic and invasive ability, with a higher expression of P-glycoprotein (P-gp), compared with the parental cells. This phenomenon was at least partially due to EMT and the appearance of MDR in sorafenib-resistant HCC cells. Moreover, MDR was a downstream molecular event of EMT. Silencing Snail with siRNA blocked EMT and partially reversed the MDR, thereby markedly abolishing invasion and metastasis in sorafenib-resistant HCC cells, but silencing of MDR1 had no effect on the EMT phenotype. Additionally, HCC parental cells that were stably transfected with pCDNA3.1-Snail exhibited EMT and MDR. Two sorafenib-resistant HCC cell lines, established from human HCC HepG2 and Huh7 cells, were refractory to sorafenib-induced growth inhibition but were sensitive to MK-2206, a novel allosteric AKT inhibitor. Thus, the combination of sorafenib and MK-2206 led to significant reversion of the EMT phenotype and P-gp-mediated MDR by downregulating phosphorylated AKT. These findings underscore the significance of EMT, MDR and enhanced PI3K/AKT signaling in sorafenib-resistant HCC cells.

## Introduction

Hepatocellular carcinoma (HCC) is the most common histological type of primary liver cancer and the second largest cause of cancer-related death in men worldwide [1]. Surgical resection and traditional chemotherapy are the typical forms of treatment for patients with HCC. However, the overall prognosis of patients with liver cancer is poor, and only a minority of HCC patients are eligible for surgical resection due to late stage diagnosis [2]. Sorafenib is a multikinase inhibitor with antiangiogenic and antiproliferative effects and the only drug that is clinically approved for patients with advanced HCC [3]. The major target of sorafenib is the serine/threonine kinase Raf-1, which is involved in the Raf/mitogen-activated protein kinase (MAPK)/extracellular signaling-regulated kinase (ERK) pathway [4]. Sorafenib exerts potent inhibitory activity against cell proliferation, invasion, metastasis and multi-drug resistance (MDR) by inhibiting MAPK signaling in HCC [5,6]. However, this promising treatment has demonstrated limited survival benefits (2.8 months) with very low response rates (2–3%) [3,4], and some advanced HCC patients under long-term treatment with sorafenib have enhanced tumour growth or distant metastasis [7], indicating that resistance to sorafenib is common in HCC.

Several studies have claimed that epithelial-mesenchymal transition (EMT) is involved in shorter disease-free survival as well as chemoresistance in HCC [8–10]. EMT, a developmental process that involves the loss of epithelial cell markers and the acquisition of mesenchymal cell characteristics, has important roles in the development of the invasive and metastatic potential of HCC [11]. Characteristic downregulation of E-cadherin is regarded as the key step of EMT, and the zinc-finger transcriptional repressors Snail, Slug and Twist, which bind to E-boxes of the E-cadherin promoter and suppress its transcription in response to upstream signaling, are the most prominent suppressors of E-cadherin transcription [12]. In addition, the Snail transcription factor plays a pivotal role in the expression of mesenchymal markers such as Vimentin and matrix metalloproteinases (MMP-2, 9) in HCC cells [13]. These studies suggest that expression of the Snail transcription factor is an important step leading to invasion, metastasis and HCC progression. In a previous report, sorafenib was shown to exert potent inhibitory activity against EMT by inhibiting Snail expression via the MAPK signaling pathway in HCC cells [5], but it has also been reported that, in sorafenib-resistant HCC cells, EMT was accompanied by activation of the phosphoinositide 3-kinase (PI3K)/AKT pathway [14], indicating that the complicated role of EMT in sorafenib resistance is far from clear. Emerging evidence suggests that MDR in human HCC is associated with the activation of the PI3K/AKT pathway [15]. MDR, a phenotype of cancer cells, is a condition in which cancer cells acquire resistance to multiple different drugs, which have virtually nothing in common, and it has become a major challenge considering the irreplaceable role of chemotherapeutic intervention in cancer treatment [16]. Additional research has shown that EMT is associated with MDR in HCC, and the expression of P-glycoprotein (P-gp), which is encoded by the multidrug resistance protein 1 (MDR1) gene, is associated with increased cell migration and invasion in HCC [17,18]. However, the relationship between EMT and MDR in sorafenib-resistant HCC cell lines has rarely been reported. In the present study, we tested and verified that EMT and MDR appear in sorafenib-resistant HCC cells and demonstrated MDR-relevant mechanisms of EMT are closely related to the PI3K/AKT/Snail pathway. Moreover, MK-2206 in combination with sorafenib significantly reverts the EMT phenotype and P-gp-mediated MDR.

## Materials and methods

### Cell culture, antibodies, and reagents

Human HCC HepG2 cells were obtained from the American Type Culture Collection (ATCC, Manassas, VA, USA), and Huh7 cells were obtained from the Chinese Academy of Sciences Cell Bank (Shanghai, China). Cells were cultured at 37°C in Dulbecco’s modified Eagle’s medium (DMEM) supplemented with 10% FBS (Gibco BRL), under 5% CO_2_ in an incubator. The primary antibodies (Abs) against AKT, p-AKT (Ser473), glycogen synthase kinase (GSK)-3β and phosphorylated GSK3β (p-GSK3β) were purchased from Cell Signaling Technology (Danvers, USA). The Abs against E-cadherin, Vimentin, Snail, MMP-9 and P-gp were purchased from Santa Cruz Biotechnology (CA, USA). The anti-β-actin Ab was purchased from Beijing Zhongshan Biotechnology Co., Ltd. (Beijing, China). Sorafenib was purchased from Jinan Trio Pharmatech Co., Ltd. (Jinan, China). 8-[4-(1-aminocyclobutyl)phenyl]-9-phenyl-1,2,4-triazolo[3,4-f][1,6]naphthyridin-3(2H)-one dihydrochloride (MK-2206) was from Shanghai Biochempartner Co., Ltd. (Shanghai, China). Sorafenib and MK-2206 were dissolved in dimethyl sulfoxide to make a stock solution of 100 mmol/L, and 100 mmol/L was used for in vitro assays. The first strand cDNA synthesis kit, real-time PCR kits, Real-Time Polymerase Chain Reaction (RT-PCR) kits, polymerase chain reaction (PCR) kit, restriction enzymes, and Pfu DNA polymerase were purchased from Ta Ka Ra Biotechnology Co., Ltd. (Dalian, China).

### Establishment of sorafenib-resistant cells

Sorafenib-resistant cells were established as previously described by us [19]. Briefly, cells were cultured in 6-well plates at 1×10^4^ cells/well and incubated with sorafenib at a concentration just below their respective IC_50_. The concentration of sorafenib was slowly increased by 0.25 μmol/L per week. After 6 to 7 months, two sorafenib-resistant cell lines were obtained, termed HepG2-SR and Huh7-SR, and were continuously cultured in the presence of sorafenib to maintain the acquired resistance.

### Cell viability assay

Cell viability was determined using a 3-(4,5-dimethylthiazole-2-yl)-2.5-diphenyl tetrazolium bromide (MTT) (Beyotime Institute of Biotechnology, Shanghai, China) assay. HCC cells were plated at 5×10^3^/well in 96-well, flat-bottomed plates in DMEM with 10% FBS and incubated overnight at 37°C in a humidified incubator containing 5% CO_2_. Twenty-four hours later, cells were treated with various concentrations of sorafenib in 5% FBS-supplemented DMEM for an additional 48 h. Controls received dimethyl sulfoxide (DMSO) vehicle at a volume equal to that of the drug-treated cells. After 48 h, 20 μl of 0.5 mg/ml MTT was added to each well, followed by incubation for 4 h at 37°C. Supernatants were removed from the wells, and the formazan crystals were dissolved in 200 μl/well DMSO. Absorbance at 570 nm was determined on a plate reader. Each assay was performed three times, and the average results were calculated.

### Transfection of siRNA

All siRNA duplexes were purchased from Gemma pharmaceutical technology co., Ltd. (Shanghai, China), and their targeted genes are shown in Table 1 [20, 21, 22]. Namely, siRNA targeting Snail (Snail-siRNA), siRNA targeting MDR-1 (MDR1-siRNA) and a non-targeting control siRNA (siNegative) were used. The siRNA transfection methods have been previously described in detail [22, 23]. Briefly, HepG2-SR cells (5×10^5^ per well) were cultured in six-well plates for 24 h. Cells were grown to 60% to 70% confluence and transfected with the Snail-siRNA and/or MDR1-siRNA at a final concentration of 0.1 mmol/L using Lipofectamine2000 (Invitrogen Co., Ltd. Shanghai, China) in serum-free medium according to the manufacturer's instructions. Control cells were incubated with transfection reagent or the control nonspecific siRNA. After 24 h, then subjected to the assays. To verify the siRNA efficacy, cells were lysed and the expression of the relevant proteins was analysed by western blotting.

**Table 1:** The siRNAs used in this study and their targeted genes.

Gene	Gen Bank no.	Strands	Reference
Snail	NM_005985.2	sense strand: 5'-GCGAGCUGCAGGACUCUAATT-3'	[20]
		antisense strand: 5'-UUAGAGUCCUGCAGCUCGCTT-3'	
P-gp	NM_000927	sense strand: 5'-GGCUGGACAAGCUGUGCAUGG-3'	[21]
		antisense strand: 5'-AUGCACAGCUUGUCCAGCCAA-3'	
Control	_	sense strand: 5' -UUCUCCGAACGUGUCACGU-3'	[22]
		antisense strand: 5'-ACGUGACACGUUCGGAGAA-3'	

### Construction of the pCDNA3.1-Snail eukaryotic expression vector and transfection

RNA was extracted from the tumour tissue using TRIzol (Beijing Solar Biotechnology Co., Ltd.). A total of 2 μg of RNA was used to synthesize the cDNA, which was used as a template. The Snail gene was then amplified with Pfu (NM_005985.2). The primer pairs were 5’-TTCCTGAGCTGGCCTGTCTG-3’ (forward) and 5’-TGGCCTGAGGGTTCCTTGTG-3' (reverse) [24]. After A was attached to the end of the PCR product, it was ligated with a T vector to construct the pUCm-T-Snail vector. The T/A Cloning kit was purchased from Shanghai (Sangon Biological Engineering Technology & Services Co., Ltd. Shanghai, China). The final digestion product was ligated to pCDNA3.1 (-) (Invitrogen Co., Ltd. Shanghai, China) using BamHI/HindIII to construct the pCDNA3.1-Snail eukaryotic expression vector, and vector construction was confirmed by enzymatic digestion sequencing [25]. HepG2, HepG2-SR and Huh7 cells were grown to 80% confluence, and the cells were transfected with pCDNA-Snail and control plasmid pCDNA3.1(-) using liposome transfection methods and named HepG2/Snail, HepG2/pcDNA, HepG2-SR/Snail, HepG2-SR/pcDNA, Huh7/Snail, and Huh7/pCDNA. A total of 48 h later, rapid screening was performed with 500 μg/ml G418 (Sigma, Shanghai, China). Media were exchanged every 3-4 days. When cell death discontinued and a few cells underwent division and proliferation began to increase, 200 μg/ml G418 was used to maintain screening (approximately 12-14 days). Cells were digested in the original cell culture bottles when positive cell clones were grown to a certain number. Cells were processed after reaching 100% confluence. One week later, the cells were used in the experiments.

### Cell migration and invasion assays

Cell migration was examined using a Boyden chamber method and polycarbonate membranes with an 8-μm pore size (Corning, Costar, Cambridge, MA, USA). Cells (5×10^4^) were seeded the upper chamber with 200 μl of serum-free medium, and the upper chambers were placed into the lower chambers of 24-well culture dishes containing 600 μl of DMEM that contained 10% FBS. After 48 h, the media in the upper chambers were aspirated and the non-migrated cells on the inner sides of the membranes were removed using a cotton swab. The cells that had migrated to the outer side of the membranes were fixed with 4% paraformaldehyde for 30 minutes and stained with 0.1% crystal violet (Beyotime Institute of Biotechnology, Shanghai, China) for 10 minutes, and then, the number of migrated cells were counted in four fields under ×100 magnification per 1 chamber; 3 chambers were used in 1 experiment. The cell invasion assay was performed similarly, except 50 μl of Matrigel (BD, Biosciences, USA) diluted 1:6 with serum-free medium was added to each well and dried for 2 hours at room temperature before cells (2×10^5^) were seeded onto the membrane.

### Western blot analysis

Equal amounts of the protein extracts (40 μg, nuclear or total extract) were boiled for 10 min in sample buffer and subjected to 10% SDS-polyacrylamide gel electrophoresis and then transferred to polyvinylidene difluoride (PVDF) membranes. The membranes were blocked with 10% skim milk in PBST for 1 h at room temperature and then probed with primary antibodies against AKT, p-AKT, GSK-3β, p-GSK3β, E-cadherin, vimentin, Snail, MMP-9, P-gp and β-actin for 2 h at room temperature followed by incubation overnight. After incubation in the primary antibody, the membranes were washed three times in PBST buffer and incubated with secondary antibody for 1 h at room temperature. The antibody—antigen complexes were visualized after incubation with 2 ml of an ECL plus detection system for 5 min at room temperature and then exposed to X-OMAT film. β-actin was used as the loading control. All the experiments were performed at least in duplicate.

### Real-time reverse transcription PCR

After 48 h of transfection, total RNA was extracted from transfected and parental cells using TRIzol (Beijing Solar Biotechnology Co., Ltd.) reagent according to the manufacturer’s instructions [26]. The primers used for amplification of human genes are shown in Table 2. The PCR array assays were performed as previously described [27]. Briefly, the reverse transcription products were loaded onto the Taq Man array for real-time PCR amplification using an MX3000P real-time PCR system (Stratagen, USA). The amplification specificity was confirmed by the melting curves. Relative mRNA levels of Snail and P-gp were calculated based on the Ct values and normalized using β-actin expression, according to the equation: 2^−ΔCt^[ΔCt = Ct _target gene_-Ct_β-actin_]. All experiments were performed in triplicate.

**Table 2:** The primers and reaction conditions for real-time PCR.

Gene	Primer	Annealing temperature (°C)	Production (bp)	Reference
Snail	forward: 5'-TTCCTGAGCTGGCCTGTCTG-3'	58°C	799	[24]
	reverse: 5'-TGGCCTGAGGGTTCCTTGTG-3'			
P-gp	forward: 5'-GCCCTTGGAATTATTTCTTT -3'	54°C	172	[25]
	reverse: 5'-TGGGTGAAGGAAAATGTAAT -3'			
β-actin	forward: 5'-TACCTCATGAATAGCCTCACC-3'	60°C	126	[27]
	reverse: 5'-TTTCGTGGATGCCACAGGAC -3'			

### Animal experiments

Six to 8-week-old male nude BALB/c mice (H-2b) were obtained from SLAC laboratory Animal Co., Ltd. (Shanghai, China). This study had been approved (permit SYXK20020009) by the Animal Ethics Committee of Harbin Medical University, in compliance with the Experimental Animal Regulations by the National Science and Technology Commission, China. The experimental protocol has been described previously [19, 28]. Briefly, Huh7, Huh7-SR or Huh7/Snail cells (5 × 10^6^) were inoculated subcutaneously into mice. To maintain the sorafenib-resistant ability of injected Huh7-SR cells, sorafenib was administrated to mice injected with Huh7-SR cells at a dose of 15 mg/kg every three days. When the Huh7, Huh7-SR and Huh7/Snail tumors reached ~ 100 mm^3^, sorafenib was suspended in an oral vehicle containing Cremophor (Sigma-Aldrich), 95% ethanol and water in a ratio of 1:1:6 [19], and administrated orally at a dose of 30 mg/kg by gavage daily. The tumor volumes were measured every 3 days.

### Statistical analysis

Data are shown as the mean ± standard deviation. Data were pooled from at least three independent experiments to avoid possible variation between the cell cultures. For statistical analyses, Student’s t-test was employed, and P<0.05 was considered statistically significant.

## Results

### EMT and MDR appears in sorafenib-resistant HCC cells

**Fig 1:**
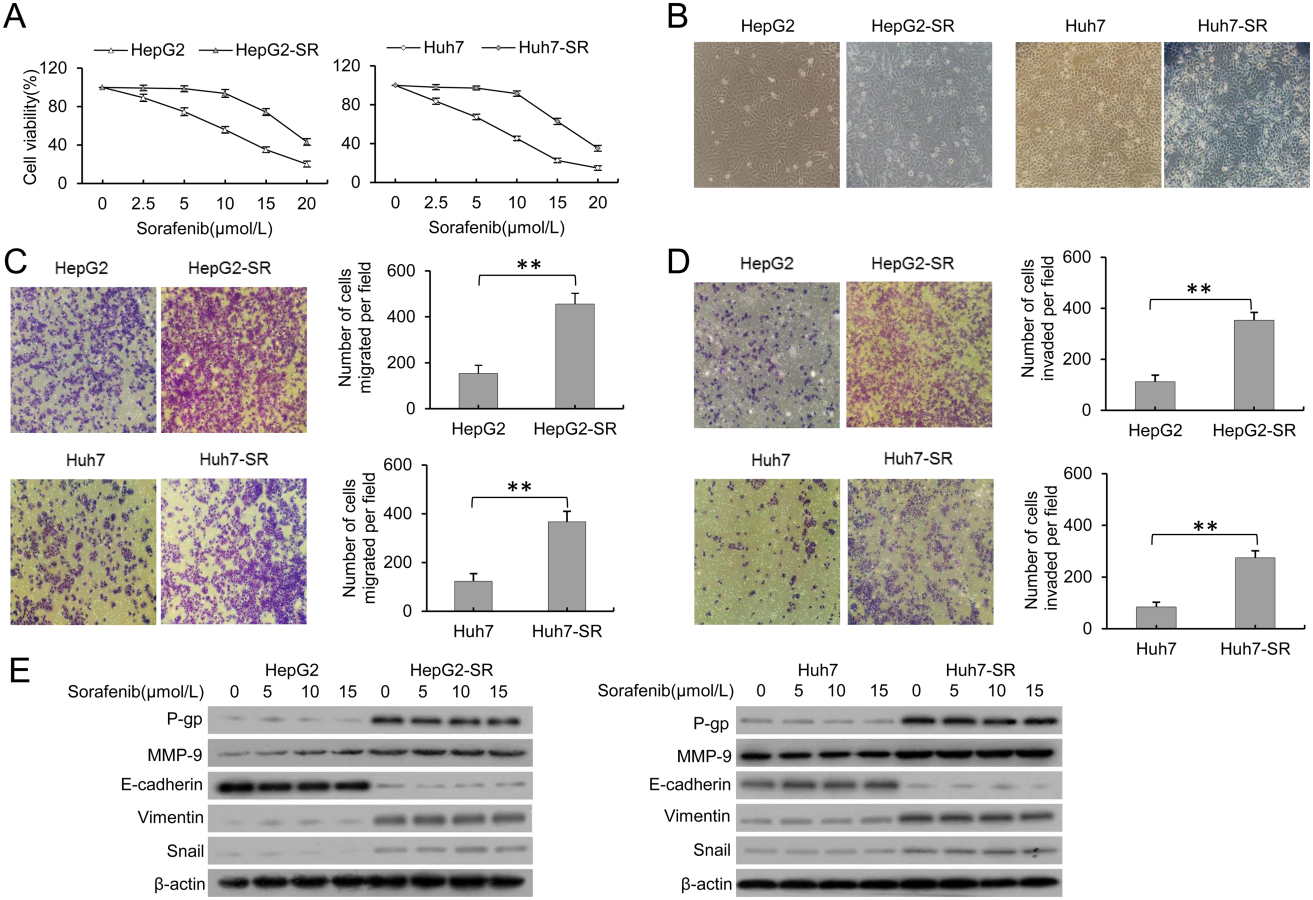
EMT and MDR appears in sorafenib-resistant HCC cells. A, The sorafenib-resistant HepG2-SR and Huh7-SR cells and parental HepG2 and Huh7 cells were incubated with increasing concentrations of sorafenib for 48 hours. Cell viability (%) was assessed and compared with the corresponding untreated cells. B, Morphological changes were evaluated in and sorafenib-resistant cells under ×400 magnification. C, Migration of HCC cells was measured by with a Transwell assay. D, Invasion of HCC cells was also measured with a Transwell assay. E, EMT- and MDR-related proteins, including E-cadherin, Vimentin, MMP-9, Snail and P-gp, were assessed by western blotting in parental and resistant cells. β-actin was used as a loading control in the western blot analysis. Data represent three independent experiments. ** indicates P<0.001.

As previously described, two sorafenib-resistant cell lines, HepG2-SR and Huh7-SR, were established by incubating Huh7 and HepG2 cells with increasing concentrations of sorafenib [19]. After incubation with increasing concentrations of sorafenib for 48 hours, the viability of HepG2-SR and Huh7-SR cells was significantly higher than that of the corresponding parental cells (Fig 1A). Concomitant with the appearance of the drug resistance characteristic, HepG2-SR and Huh7-SR cells underwent significant morphological changes and displayed a mesenchymal phenotype (Fig 1B). EMT is well known to be closely correlated with cancer invasion and migration [11]. We next investigated the metastatic and invasive characteristics of the cells using a Transwell assay. As shown in Fig 1C, enhanced metastatic characteristics were detected in the HepG2-SR and Huh7-SR cells compared with the corresponding parental cells. Moreover, the sorafenib-resistant cells also showed increased invasive activity (Fig 1D). Importantly, the epithelial marker E-cadherin was downregulated, and the mesenchymal markers Vimentin and MMP-9 were upregulated in the HepG2-SR and Huh7-SR cells. Snail, a zinc-finger transcriptional repressor that plays a key role in EMT-mediated tumour invasion and metastasis, was also upregulated in the HepG2-SR and Huh7-SR cells when compared to the controls. The multidrug resistance of HepG2-SR and Huh7-SR cells supported by the increased protein expression of P-gp compared with parental cells (Fig 1E).

### Silencing of Snail blocked EMT and reversed the drug resistance in sorafenib-resistant HCC cells

**Fig 2:**
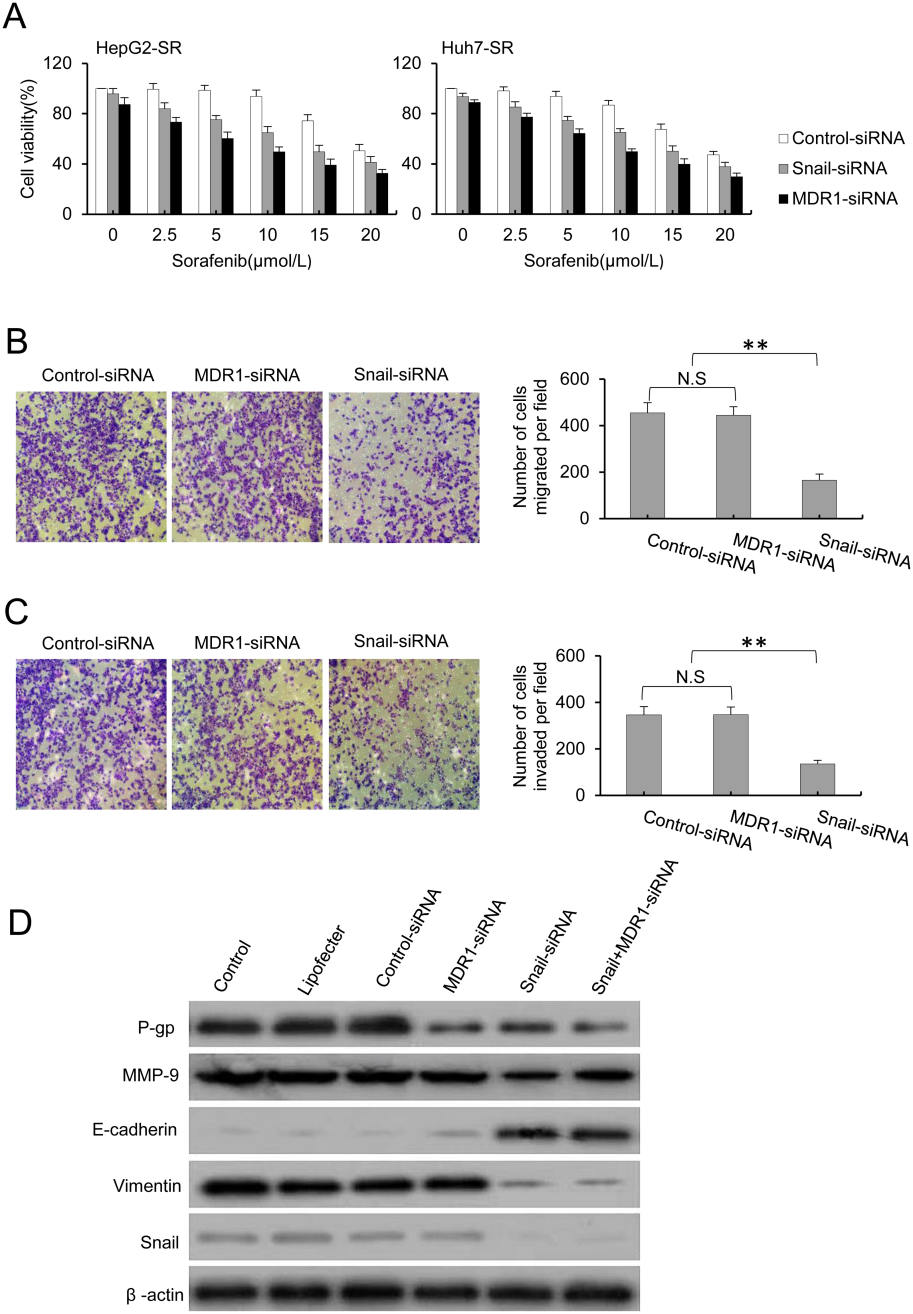
Silencing of Snail blocked EMT and reversed the drug resistance in sorafenib-resistant HCC cells. A, The sorafenib-resistant HepG2-SR and Huh7-SR cells were transfected with Snail siRNA and MDR siRNA, and cells were then incubated with increasing concentrations of sorafenib for 48 hours. Cell viability (%) was assessed and compared with the corresponding untransfected cells. B, Knockdown of snail abolished the metastatic potential of HepG2-SR cells. C, Knockdown of snail abolished the invasive ability of HepG2-SR cells. D, HepG2-SR cells were transfected with control or Snail and/or MDR1 siRNA for 24 hours, and the protein levels of EMT and MDR markers were detected by western blot. β-actin served as the loading control in the western blot analysis. Data represent three independent experiments. N.S., not significant. * indicates P<0.05, and ** indicates P<0.001.

To further explore the role of EMT in the acquired drug resistance to sorafenib, we used siRNA to silence the Snail and MDR1 genes. To investigate the effect of EMT on cell growth, HepG2-SR and Huh7-SR cells were transfected with control or Snail or MDR1 siRNA for 24 h and then further incubated with increasing concentrations of sorafenib for 48 h. Cell survival was then assessed via an MTT assay. As shown in Fig 2A, Snail or MDR1 siRNA significantly inhibited the cell viability of drug-resistant cells compared with control cells. Moreover, when in combination with sorafenib, Snail or MDR1 siRNA significantly strengthened the sorafenib-induced cell growth inhibition. This finding indicated that blocking EMT reversed drug resistance to sorafenib in HCC cells. To further investigate the invasion and migration ability, HepG2-SR cells were transfected with Snail siRNA or MDR1 siRNA for 24 h, and then, the cells were harvested for a assay. As shown in Fig 2B and 2C, silencing of Snail significantly downregulated the invasion and migration of HepG2-SR cells. However, silencing of MDR1 did not have the same effect on the invasion and migration ability. To explore the molecular mechanism of Snail and MDR1 silencing in reverting the drug resistance to sorafenib, the protein levels of EMT and MDR markers were detected by western blot. As shown in Fig 2D, sorafenib-resistant cells showed an EMT phenotype, represented by the upregulation of Vimentin, MMP-9, and Snail and the downregulation of E-cadherin, and MDR characteristics, with P-gp upregulation compared to parental cells. Silencing of Snail resulted in upregulation of E-cadherin and downregulation of Vimentin, MMP-9, and Snail, which represented a reversed EMT phenotype. Moreover, silencing of Snail simultaneously downregulated P-gp expression. However, silencing of MDR1 had no effect on the EMT phenotype. When the MDR1 siRNA was used to downregulate P-gp, the role of Snail siRNA in reversing the EMT phenotype was not blocked. These results indicate that Snail depletion abolished the mesenchymal transformation and reversed MDR by inhibiting invasion and migration. Moreover, the MDR was downstream of the molecular events of EMT.

### HCC parental cells stably transfected with pCDNA3.1-Snail exhibited EMT and MDR

**Fig 3:**
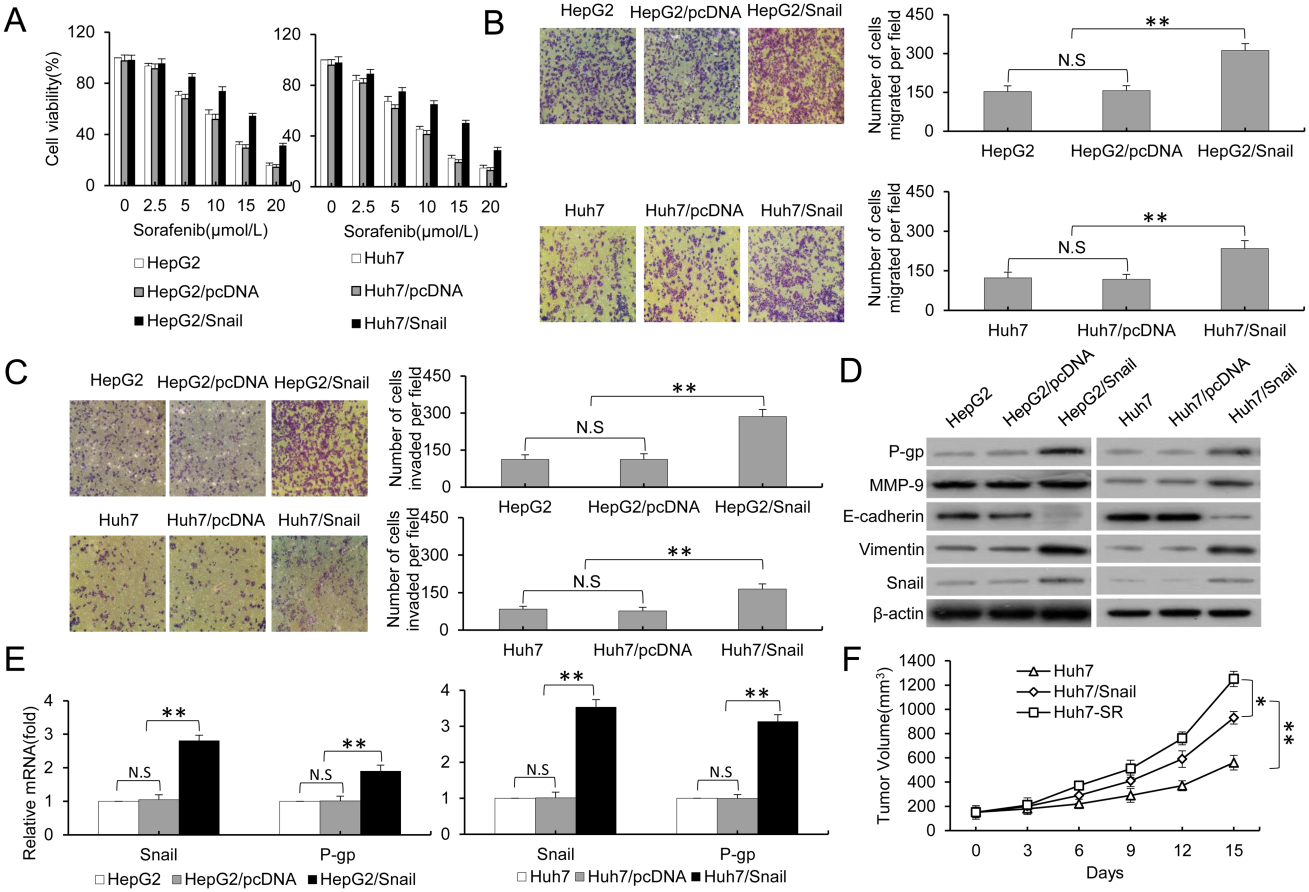
HCC parental cells stably transfected with pCDNA3.1-Snail exhibited EMT and MDR. A, Untransfected HepG2 and Huh7 cells or cells transfected with control or pCDNA3.1-Snail were incubated with increasing concentrations of sorafenib for 48 hours. Cell viability (%) was assessed and compared with the corresponding control cells. B, High expression of Snail upgraded the metastatic ability of HCC cells. C, High expression of Snail upgraded the invasion potential of HCC cells. D, The protein expression profiles of untransfected HepG2 and Huh7 cells or cells transfected with control or pCDNA3.1-Snail were detected by western blot analysis. β-actin served as the loading control in the western blot analysis. E, Cells from (D) were subjected to real-time PCR to detect the expression of Snail and the multidrug resistance protein P-gp; the results were normalized to β-actin. F, Subcutaneous tumors formed from Huh7, Huh7-SR and Huh7/Snail cells were established in mice, which received 30 mg/kg sorafenib for 15 days as described in MATERIALS AND METHODS. The sizes (mm^3^) of tumors were recorded. Data represent three independent experiments. N.S., not significant. * indicates P<0.05, and ** indicates P<0.001.

Next, we further explored the relationship between EMT and MDR and the role in drug resistance to sorafenib. As previously described, we constructed a pCDNA3.1-Snail expression vector and established stably transfected HepG2/snail and Huh7/snail cells. As shown in Fig 3A, the HepG2/snail and Huh7/snail cells showed the drug resistant characteristic. HepG2/snail and Huh7/snail cells and the corresponding parental cells were incubated with increasing concentrations of sorafenib for 48 hours and then subjected to an MTT assay. HepG2/snail and Huh7/snail cells showed significantly higher cell viability compared to the control cells at the same concentrations of sorafenib. Even when the concentration of sorafenib reached 20 μmol/L, the viability of HepG2/snail and Huh7/snail cells still remained at 31.2% and 25.1%, respectively, whereas almost all of the HepG2 and Huh7 cells were dead. Next, the half-maximal inhibitory concentration (IC50) of sorafenib in the above cells was determined. The IC50 values of HepG2/snail and HepG2 cells were 16.4 μmol/L and 10.1 μmol/L, and the IC50 values of Huh7/snail and Huh7 cells were 15.2 μmol/L and 8.7 μmol/L. Moreover, the HepG2/snail and Huh7/snail cell lines showed a higher metastatic ability and invasion potential compared with parental cells (Fig 3B and 3C). Furthermore, the expression of EMT- and MDR-related proteins was detected by western blot. As shown in Fig 3D, E-cadherin was downregulated, and Vimentin, Snail, MMP-9 and P-gp were upregulated in the HepG2/snail and Huh7/snail cells stably transfected with pCDNA3.1-Snail. RT-PCR further confirmed the increased mRNA expression levels of the transcription factor Snail and the multidrug resistance protein P-gp in the HepG2/Snail and Huh7/Snail cells (Fig 3E). The above in vitro findings were further supported by an in vivo study in mice that investigated the effects of sorafenib on the growth of tumors formed from Huh7, Huh7-SR and Huh7/Snail cells. As shown in Fig 3F, the Huh7 tumors were significantly smaller than the Huh7-SR and Huh7/Snail tumors. These results indicated that HCC parental cells stably transfected with pCDNA3.1-Snail exhibited EMT and MDR, which mediated the drug resistance to sorafenib.

### Activation of the PI3K/AKT signaling pathway is associated with the EMT phenotype and P-gp-mediated multidrug resistance in sorafenib-resistant HCC cells

**Fig 4:**
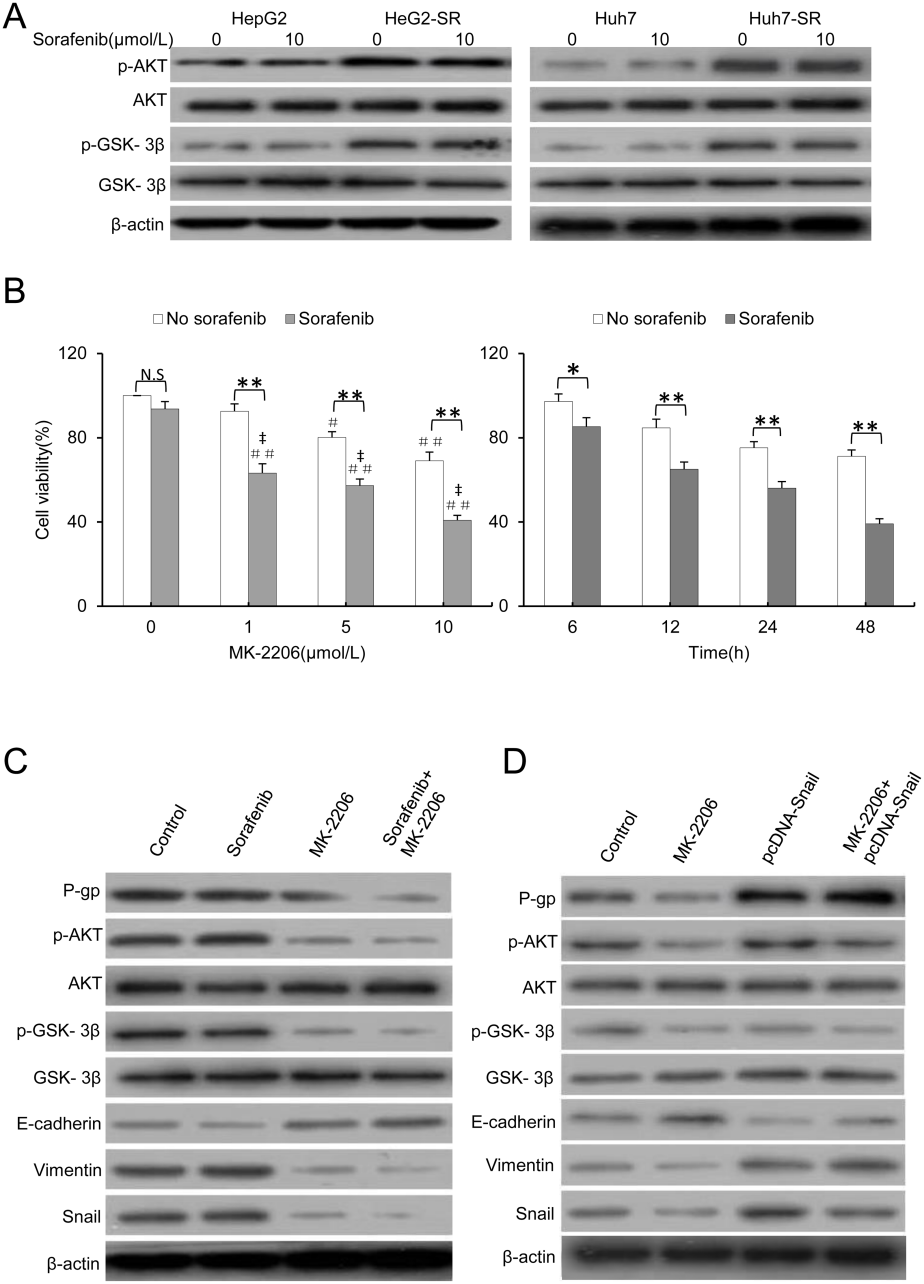
Activation of the PI3K/AKT signaling pathway is associated with the EMT phenotype and P-gp-mediated multidrug resistance in sorafenib-resistant HCC cells. A, The sorafenib-resistant cells and parental cells were incubated with 0 or 10 μmol/L sorafenib for 48 hours. The protein expression profiles were detected by western blot analysis; β-actin served as the loading control. B, HepG2-SR cells were exposed for 48 hours to different concentrations of MK-2206 or 10 μmol/L MK-2206 for different periods of time in the presence or absence of sorafenib (10 μmol/L). Cell viability (%) was assessed and compared with the corresponding untreated cells. C, HepG2-SR cells were incubated with 10 μmol/L MK-2206 and/or 10 μmol/L sorafenib for 48 hours. The protein expression profiles were detected by western blot analysis; β-actin served as the loading control. D, HepG2-SR or HepG2-SR/Snail cells were incubated with 0 or 10 μmol/L MK-2206 for 48 hours. The protein expression profiles were detected by western blot analysis; β-actin served as the loading control. The data represent three independent experiments. N.S., not significant. * indicates P<0.05, and ** P<0.001. # indicates P<0.05 and ## indicates P<0.001 vs. untreated control; ‡ indicates P <0.001 vs. sorafenib alone.

EMT and MDR are dynamic processes triggered by stimuli from the microenvironment. Several important signaling pathways, including the MAPK and PI3K/AKT pathways, have been reported to contribute to the EMT phenotype and P-gp-mediated MDR [5, 6, 15, 17,]. In our previous study, increased p-AKT was responsible for resistance to sorafenib, and specific inhibition of AKT reversed the drug resistance to sorafenib [19]. Therefore, in the current study, we further examined the role of the PI3K/AKT pathway in EMT- and MDR-mediated drug resistance to sorafenib. As shown in Fig 4A, in sorafenib-resistant cells, AKT was activated. In addition, a downstream molecule of AKT, GSK-3β, which mediates the stabilization of endogenous Snail, was also phosphorylated [29, 30]. Furthermore, MK-2206, a highly selective non-ATP-competitive allosteric inhibitor of AKT was used to inhibit AKT [31]. As shown in Fig 4B, MK-2206 had a synergistic effect with sorafenib in reducing the viability of HepG2-SR cells in a dose- and time-dependent manner. The coefficient of drug interactions (CDIs) for HepG2-SR cells treated with 1, 5, or 10 μmol/L of MK-2206 in the presence of sorafenib (10 μmol/L) were 0.72, 0.65, or 0.68, respectively, indicating that the synergistic effects were significant. We next examined whether blocking AKT could reverse the EMT phenotype and MDR in drug-resistant HCC cells. HepG2-SR cells were incubated with 10 μmol/L MK-2206 and/or 10 μmol/L sorafenib for 48 h and then subjected to an immunoblotting analysis. As shown in Fig 4C, MK-2206 inhibited the activation of p-AKT and the downstream phosphorylation of GSK-3β, while the protein expression of E-cadherin was upregulated and the expression of Snail, Vimentin and P-gp were downregulated, indicating reversion of the EMT phenotype and P-gp-mediated multidrug resistance in MK-2206-treated sorafenib-resistant cells. MK-2206 in combination with sorafenib also significantly reversed the EMT phenotype and P-gp-mediated MDR. Moreover, overexpression of Snail alone significantly upregulated the expression of Snail and P-gp protein, but did not affect AKT and GSK-3β expression; When Snail overexpression was combined with MK-2206, Snail overexpression significantly reversed MK-2206-induced E-cadherin upregulation and Snail, Vimentin and P-gp downregulation (Fig 4D). These results indicate that an AKT inhibitor reverses the acquired resistance to sorafenib by reversing MDR in an EMT-dependent manner via downregulation of p-AKT.

## Discussion

Since the clinical approval of sorafenib, it has become the new standard therapy for patients with advanced HCC [3]. However, this promising treatment has demonstrated limited survival benefits with very low response rates [3, 4]. Furthermore, long-term sorafenib treatment in advanced HCC patients may have enhanced tumour growth or distant metastasis [7]. In the present study, two sorafenib-resistant HCC cell lines were obtained from long-term exposure to high-dose sorafenib, and the sorafenib-resistant HCC cells exhibited EMT and MDR. Moreover, the MDR was a downstream molecular event of EMT, and MK-2206, an AKT-specific inhibitor, in combination with sorafenib significantly reversed the EMT phenotype and P-gp-mediated MDR in sorafenib-resistant HCC cells.

Recently, accumulating evidence has shown that EMT is involved in sorafenib resistance in HCC cells [32]. Snail, an EMT-inducing transcription factor, has a central role in cancer invasion, and overexpression of Snail increases the recurrence rate and is a marker for poor survival in HCC patients [33]. In a recent study, EMT was shown to have a critical role in drug resistance to several cytotoxic chemotherapeutic and molecular targeted drugs, including sorafenib, in HCC [7, 9]. However, the exact molecular mechanisms of EMT in drug resistance to sorafenib are far from clear. P-gp is the most common member of the ATP binding cassette (ABC) family of transporters. It is known that MDR also intervenes in the acquired resistance to sorafenib [34]. However, whether P-gp plays a role in the resistance to sorafenib in HCC urgently requires clarification. In our previous report, two sorafenib-resistant cell lines from HepG2 and Huh7 cell lines, named HepG2-SR and Huh7-SR, were established and were shown to have obvious sorafenib-resistant characteristics [19]. Herein, we have demonstrated that sorafenib-resistant HCC cells show enhanced migration and invasion abilities compared with the corresponding parental cells, in accordance with a previous report [14]. In this study, hallmarks of EMT and MDR were upregulated in sorafenib-resistant HCC cells compared to the corresponding parental cells, which indicates EMT and MDR activation.

Several studies have reported that EMT is related to the acquisition of the MDR phenotype [35, 36]. One report showed that breast cancer cells with MDR displayed enhanced metastatic activity, and they overexpressed N-cadherin, vimentin and the EMT-inducing transcription factors Slug, Twist and ZEB1/2 [37]. Other research has shown that EMT plays a critical role in Adriamycin-based chemotherapies, and this role has been attributed to its ability to induce MDR in breast cancer cells. This research showed that only cells undergoing EMT displayed enhanced invasion and MDR [38]. Moreover, a recent study showed that HCC cells that underwent EMT displayed enhanced invasiveness and MDR during hypoxia [17]. Here, to explore whether crosstalk between EMT and MDR is involved in the acquired drug resistance to sorafenib in HCC, siRNAs were used to silence the Snail and MDR1 genes, and then, the EMT phenotype and drug resistance were examined. As shown in Fig 2, silencing of Snail reversed the EMT phenotype and partially reversed the MDR, and it markedly abolished invasion and migration, as evidenced by the upregulation of E-cadherin and the downregulation of Vimentin, MMP-9, Snail and P-gp. Moreover, silencing of Snail simultaneously inhibited the migration and invasion of sorafenib-resistant cells. MDR1 siRNA was used to downregulate P-gp, the effect of Snail siRNA on reversion of the EMT phenotype was not blocked. These results suggested that MDR was a downstream molecular event of EMT during the development of drug resistance to sorafenib in HCC. To further confirm the above conclusion, a pCDNA3.1-Snail expression vector was constructed, and stably transfected HepG2 and Huh7 cells were constructed. Snail plays an important role in HCC cells in inducing EMT by downregulating cell adhesion molecules such as E-cadherin by binding several E-boxes located in the E-cadherin promotor region [39]. Moreover, overexpression of Snail is associated with facilitated acquisition of P-gp-mediated MDR [25]. Here, we showed that the HepG2/snail and Huh7/snail cells showed apparent drug resistance to sorafenib compared to the corresponding parental cells, and this was accompanied by EMT and MDR characteristics. These results indicated that Snail-overexpressing HCC cells were more resistant to sorafenib through increased expression of P-gp.

The PI3K/AKT pathway is involved in the development and progression of HCC and is activated in 92.3% of HCC specimens [40, 41]. Sorafenib was previously reported to activate the PI3K/AKT pathway in both parental and sorafenib-resistant HCC cells, and blocking the PI3K/AKT signaling pathway enhances the efficacy of sorafenib [19, 23, 42, 43]. Sorafenib exerts potent inhibitory activity against the EMT phenotype and P-gp-mediated MDR by inhibiting MAPK signaling in HCC [5, 6], whereas the long-term exposure of liver cancer cells to sorafenib has been reported to induce resistance, EMT, increased invasion and risk of rebound growth [14]. There is crosstalk between the PI3K/AKT and MAPK/ERK pathways [40], indicating that a latent compensatory mechanism of the PI3K/AKT pathway may contribute to EMT and MDR phenotypes in sorafenib-resistant HCC cells. In our previous study, increased p-AKT was responsible for resistance to sorafenib, and specific inhibition of AKT could reverse the drug resistance to sorafenib [19]. In the current study, we further investigated the role of the PI3K/AKT signaling pathway in EMT and MDR-mediated drug resistance to sorafenib in HCC. Consistent with our previous studies, GSK-3β, a downstream molecule of AKT, was phosphorylated in sorafenib-resistant HCC cells. GSK-3β has been reported to be involved in the activation and stabilization of endogenous Snail [29, 30]. To test whether crosstalk between the AKT pathway and the MDR and EMT pathways plays a role in mediating sorafenib resistance in HCC, MK-2206, an AKT-specific inhibitor, was used to block the AKT pathway [31], and the EMT phenotype and MDR were detected by determining the protein expression of E-cadherin, Snail, Vimentin and P-gp. The results showed that MK-2206 reversed the drug resistance to sorafenib by inhibiting the activation of AKT and the downstream kinase GSK-3β. Moreover, the protein expression of E-cadherin was upregulated and Snail, Vimentin and P-gp were simultaneously downregulated, which hints that EMT and MDR were blocked in sorafenib-resistant cells. These results indicate that activation of the PI3K/AKT signaling pathway is associated with the EMT phenotype and P-gp-mediated MDR in sorafenib-resistant HCC cells.

In conclusion, the present study demonstrated that EMT and MDR appears in sorafenib-resistant HCC cells, and EMT is a major contributor to MDR in HCC. Sorafenib-resistant HCC cells, having undergone EMT due to overexpression of Snail, are prone to sorafenib resistance through increased expression of P-gp. PI3K/AKT signaling was activated in sorafenib-resistant HCC cells, and MK-2206 reversed the acquired resistance to sorafenib by reversing MDR in an EMT- dependent manner via downregulation of p-AKT. These results identified PI3K/AKT/Snail signaling as a pivotal regulator of EMT and MDR process in sorafenib-resistant HCC cells, and this mechanism may serve as a potential therapeutic target.

## Abbreviations

Abs: antibodies
CDI: coefficient of drug interaction
DMEM: Dulbecco’s modified Eagle’s medium
DMSO: dimethyl sulfoxide
EMT: epithelial–mesenchymal transition
ERK: extracellular signaling-regulated kinase
GSK-3β: glycogen synthase kinase-3β
HCC: hepatocellular carcinoma
IC50: half-maximal inhibitory concentration
MAPK: mitogen-activated protein kinase
MDR: multidrug resistance
MK-2206: 8-[4-(1-aminocyclobutyl)phenyl]-9-phenyl-1,2,4-triazolo[3,4-f][1,6]naphthyridin-3(2H)-one dihydrochloride
MMPs: matrix metalloproteinases
MTT: 3-(4,5-dimethylthiazol-2-yl)-2, 5-diphenyl-2H-tetrazolium bromide
P-gp: P-glycoprotein
PI3K: phosphoinositide 3-kinase
PVDF: polyvinylidene difluoride
